# Characteristics of the Electrocardiogram in Japanese Fabry Patients Under Long-Term Enzyme Replacement Therapy

**DOI:** 10.3389/fcvm.2020.614129

**Published:** 2021-01-14

**Authors:** Satoshi Morimoto, Ayumi Nojiri, Eiko Fukuro, Ikuko Anan, Makoto Kawai, Ken Sakurai, Masahisa Kobayashi, Hiroshi Kobayashi, Hiroyuki Ida, Toya Ohashi, Takahiro Shibata, Michihiro Yoshimura, Yoshikatsu Eto, Kenichi Hongo

**Affiliations:** ^1^Division of Cardiology, Department of Internal Medicine, The Jikei University School of Medicine, Tokyo, Japan; ^2^Department of Laboratory Medicine, The Jikei University School of Medicine, Tokyo, Japan; ^3^Department of Pediatrics, The Jikei University School of Medicine, Tokyo, Japan; ^4^Division of Gene Therapy, Research Center for Molecular Sciences, The Jikei University School of Medicine, Tokyo, Japan; ^5^Advanced Clinical Research Center, Institute of Neurological Disorders, Kanagawa, Japan

**Keywords:** Fabry disease, electrocardiogram (ECG), enzyme replacement therapy (ERT), gender difference, left ventricular hypertrophy (LVH)

## Abstract

**Objective:** An electrocardiogram (ECG) is an important tool for demonstrating cardiac manifestations in various heart diseases. The present study clarified the characteristics of ECG parameters in Japanese Fabry patients under long-term enzyme replacement therapy (ERT).

**Methods:** We analyzed the ECGs of 40 Fabry patients (male, *n* = 17; female, *n* = 23) before and after treatment with ERT. To evaluate the atrio-ventricular conduction, the PQ interval, corrected PQ and PQ minus P-wave in lead II (Pend-Q) were calculated. The QRS duration, QTc, Sokolow-Lyon index, and strain pattern were also examined.

**Results:** At the baseline, the shortening of the PQ interval, corrected PQ and Pend-Q was identified in 7.5, 25.0, and 47.5% of cases, respectively. The prolongation of QRS duration and QTc was found in 7.5 and 40.0% of cases, respectively. The strain pattern was mainly identified in female patients, irrespective of left ventricular hypertrophy (LVH). During long-term ERT, the PQ interval, corrected PQ and Pend-Q did not change significantly. The QRS duration was significantly prolonged in both genders, whereas the QTc was significantly prolonged only in male patients. A subgroup analysis revealed that the prolongation of the QRS duration and QTc only occurred in male patients with LVH and only occurred in female patients with the classical type mutation. The prevalence of the strain was significantly increased only in male patients with LVH.

**Conclusions:** These results suggest that the shortening of the Pend-Q is a specific finding in Japanese Fabry patients, and the strain pattern without LVH in female patients can be considered Fabry disease. During long-term ERT, prolongation of the QRS duration and QTc can indicate the progression of myocardial damage in male patients with LVH and in female patients with the classical type mutation.

## Introduction

Fabry disease is an inherited metabolic disorder due to a mutation in the gene that encodes lysosomal enzyme alpha-galactosidase A (GLA). The *GLA* gene mutation causes the decrease or loss of GLA activity, leading to the accumulation of substrates predominantly globotriaosylceramide (Gb3) in various organs (1, 2).

Major affected organs related to the prognosis of Fabry disease are the heart, kidney and central nervous system (3–5). Cardiac involvement is particularly important because more than half of deaths in Fabry disease patients are due to cardiovascular events (3, 6). We previously reported the beneficial effect of enzyme replacement therapy (ERT) on echocardiographic findings (7) and the characteristics of late-gadolinium enhancement in our cohort of Fabry disease patients (8). An electrocardiogram (ECG) is a very important tool for depicting the cardiac manifestations of various heart diseases.

While left ventricular hypertrophy (LVH) is the most common cardiac manifestation, the most important manifestations are the various arrhythmias, as these are what causes death (9, 10). Indeed, cardiac dysfunction (systolic and/or diastolic), bradyarrhythmia (sick sinus syndrome and atrio-ventricular block), and tachyarrhythmia (ventricular tachycardia or fibrillation) have been demonstrated in various stages of Fabry disease (11, 12). Previous studies reported that the shortening of the PQ interval is a specific finding in Fabry disease (13), but a recent report found that only 14% of cases showed PQ shortening (14). There have been few reports regarding the characteristics of ECG parameters in Japanese Fabry disease patients (15, 16). Furthermore, the changes in various ECG parameters during long-term ERT have not been presented.

In the present study, we analyzed the ECG parameters at the initiation of ERT and evaluated the changes in those parameters following long-term ERT.

## Materials and Methods

### Population

Forty adult Fabry disease patients who were followed at Jikei University Hospital participated in the present study (males, *n* = 17; females, *n* = 23). The patients were diagnosed with Fabry disease based on a deficiency in GLA activity in their white blood cells for male patients and based on a mutation analysis of the *GLA* gene for both genders. All patients had undergone ERT for at least 3 years at the time of the inclusion. The patients in the same cohort were used to evaluate the effect of ERT on echocardiographic changes during ERT (7). Because we analyzed various ECG parameters in each patient, patients with atrial fibrillation were excluded from the present study.

This study conformed to the ethical guidelines of the 2013 Declaration of Helsinki and was approved by the Ethics Committee of The Jikei University School of Medicine. Informed consent was obtained from each of the patients who were included in this study.

Neither the patients nor the public were involved in the design, or conduct, or reporting, or dissemination plans of our research.

### Treatment Strategy for ERT

ERT was started when the physician recommended the treatment strategy and the patient agreed to the treatment. Male patients were indicated for ERT when they displayed any symptoms, while ERT was recommended to female patients when they displayed any symptoms or reached 20 years old. Recently, a national guideline for Fabry disease was published, describing obvious major organ damage (renal, cardiac, and cerebrovascular complication) as the indication for ERT in female patients. However, we previously described the merits of starting ERT early, even in female patients (7). Recombinant alpha-galactosidase A formulation was administered intravenously every 2 weeks. Agalsidase alpha was infused at a dose of 0.2 mg/kg body weight, and agalsidase beta was infused at a dose of 1 mg/kg body weight.

### ECG Examinations

Twelve-lead ECGs were recorded in all patients before and after ERT. All ECGs were independently analyzed and measured the following parameters: the PQ interval, PQ interval normalized by RR interval (corrected PQ), PQ interval minus P-wave duration in lead II (Pend-Q), QRS duration, QT interval normalized by RR interval (QTc), presence of a strain pattern, and Sokolow-Lyon index to define LVH (17). The shortening of the PQ interval was defined as ≤120 ms. The corrected PQ was calculated as the PQ interval divided by the square root of the RR interval (PQ interval/√[RR interval]), and the shortening of the corrected PQ was defined as ≤144 ms. The Pend-Q was calculated as the duration from the end of the P wave to the start of the QRS complex in lead II, and the shortening of the Pend-Q was defined as ≤40 ms. The prolongation of the QRS duration was defined as >120 ms. The QTc was calculated as the QT interval divided by the square root of the RR interval (QT interval/√[RR interval]), and the prolongation of the QTc was defined as >430 ms for males and >450 ms for females (18). A strain pattern was defined as showing a downslope ST-segment depression (>0.1 mV) with a T-wave inversion in leads V4 to V5. LVH was defined as a value of ≥3.5 mV in the Sokolow-Lyon index (S in V1 + R in V5–V6).

### Echocardiographic Examinations

Echocardiography was performed on all patients before the initiation of ERT. Based on the medical records, various parameters in B mode or M mode were extracted and analyzed according to the guideline (19). To evaluate LVH, we calculated left ventricular mass according to following equation; left ventricular mass = 0.8 × 1.04 × [(IVS + Dd + PW)^3^ – (Dd)^3^] + 0.6 (g). The left ventricular mass normalized by the body surface area [left ventricular mass index, (LVMI)] (g/m^2^) was used to determine LVH. An LVMI of >115 g/m^2^ reflected LVH in males, while an LVMI of >95 g/m^2^ reflected LVH in females (19).

### Statistical Analyses

Continuous variables were presented as the mean ± standard deviation and/or median (Q1, Q3), and categorical data were expressed as numbers and percentages. For the comparison of the two data sets, Student's *t*-test was used for continuous variables, and the chi-squared test was used for categorical data. A regression analysis was performed to detect the correlation between two groups. The significance level was set at *P* < 0.05. All statistical analyses were performed using the SPSS software program (version 25; SPSS Japan Inc., Tokyo, Japan).

## Results

### Patients' Characteristics

Table 1 shows baseline characteristics of the patients. Significant gender differences were observed in the age, ERT duration and LVMI, with a younger age, longer duration of ERT, and larger LVMI being noted in male patients than in female patients. Fewer than half of the patients were treated with either agalsidase alpha or agalsidase beta; the majority of the patients were instead treated with both types of ERT during long-term follow up, partially due to the global shortage of agalsidase beta. Among male patients, 15 cases were confirmed by the presence of a pathogenic mutation. Among those with pathogenic mutations, only 2 had a late-onset type mutation, and the other 13 had a classical type mutation. Two patients who did not agree to undergo a mutation analysis were diagnosed with the classical type based on typical clinical findings (acroparesthesias, hypohidrosis, and angiokeratoma) and very low levels of GLA activity. Among female patients, 16 had a classical type mutation, and 7 had a late-onset type mutation. There were seven cases of organ damage, all in male patients. The systolic blood pressure, diastolic blood pressure, heart rate, and estimated glomerular filtration rate (eGFR) did not differ significantly by gender. The prevalence of LVH estimated by echocardiography was higher in male patients than in female patients, although there was no statistical significance.

**Table 1:** Baseline characteristics.

	**All patients**	**Male**	**Female**
*N*	40	17	23
Age	35.5 ± 13.0	30.4 ± 9.7	39.2 ± 14.0^*^
[Median (Q1,Q3)]	33 (26.25, 41)	30 (25, 37.5)	40 (28, 50)
ERT duration (year)	9.0 ± 3.5	11.6 ± 2.4	7.1 ± 2.9^*^
[Median (Q1,Q3)]	10 (6.25, 11)	11 (10.5, 13)	7 (4,10)
**Type of ERT**			
Alpha	10	1	9
Beta	9	2	7
Beta → Alpha	13	8	5
Beta → Alpha → Beta	7	5	2
Beta → Alpha → Chaperon	1	1	0
**Phenotype/Genotype**			
Classical	31	15	16
Late-onset	9	2	7
**Comorbidity**			
Heart failure	1	1	0
End-stage renal disease	5	5	0
Stroke	1	1	0
Systolic BP (mmHg)	116.4 ± 13.9	120.6 ± 13.7	113.8 ± 13.6
Diastolic BP (mmHg)	69.3 ± 12.2	70.6 ± 13.0	68.9 ± 12.0
Heart rate (bpm)	75.2 ± 14.5	74.5 ± 16.0	75.4 ± 12.4
eGFR (ml/min/1.73 m^2^)	92.5 ± 31.8	102.1 ± 42.7	87.7 ± 20.0
LVMI (g/m^2^)	91.4 ± 26.5	107.5 ± 27.4	79.5 ± 18.8^*^
Prevalence of LVH (by echocardiography)	11 (27.5%)	6 (35.3%)	5 (21.7%)

*Alpha, agalsidase alpha; Beta, agalsidase beta; BP, blood pressure; eGFR, estimated glomerular filtration rate; ERT, enzyme replacement therapy; LVH, left ventricular hypertrophy; LVMI, left ventricular mass index*.

* *p < 0.05 comparison between male and female patients*.

### Baseline ECG Parameters

Table 2 shows the various ECG parameters at the initiation of ERT. Parameters of atrio-ventricular conduction (PQ interval, corrected PQ, and Pend-Q) did not differ significantly between male and female patients. First-degree atrio-ventricular block was observed in one male patient, and there were no patients with second- or third-degree atrio-ventricular block. Among the parameters of repolarization, the QRS duration was significantly longer in male patients than in female patients, while the QTc tended to be longer in female patients than in male patients despite no statistical significance (*P* = 0.059). There were no patients who showed bundle branch block. For the estimation of LVH using the ECG, the Sokolow-Lyon index was significantly larger in male patients than in female patients, reflecting a greater prevalence of LVH criteria in male patients than in female patients. The prevalence of a strain pattern was higher in female patients than in male patients, although the statistical significance was marginal (*P* = 0.055).

**Table 2:** Baseline ECG parameters.

	**All patients**	**Male**	**Female**
PQ interval (ms)	156.1 ± 23.4	162.4 ± 27.1	151.4 ± 19.6
Corrected PQ (ms)	157.0 ± 24.7	161.6 ± 27.5	153.6 ± 22.4
Pend-Q (ms)	4.9 ± 1.7	5.0 ± 1.9	4.8 ± 1.6
QRS duration (ms)	98.0 ± 16.6	104.1 ± 15.1	93.3 ± 16.4^*^
QTc (ms)	432.9 ± 31.0	422.2 ± 29.3	440.8 ± 30.4
Sokolow-Lyon (mV)	3.8 ± 1.3	4.8 ± 1.2	3.1 ± 0.9^*^
Prevalence of LVH (by ECG criteria)	22 (55.0%)	15 (88.2%)	7 (30.4%)^*^
Prevalence of strain	8 (20.0%)	1 (5.9%)	7 (30.4%)

*Corrected PQ, PQ interval divided by the square root of RR interval; Pend-Q, PQ interval minus P-wave duration in lead II; QTc, QT interval divided by the square root of RR interval*.

* *p < 0.05 comparison between male and female patients*.

### ECG Parameters Plotted Against LVMI

**Figure 1:**
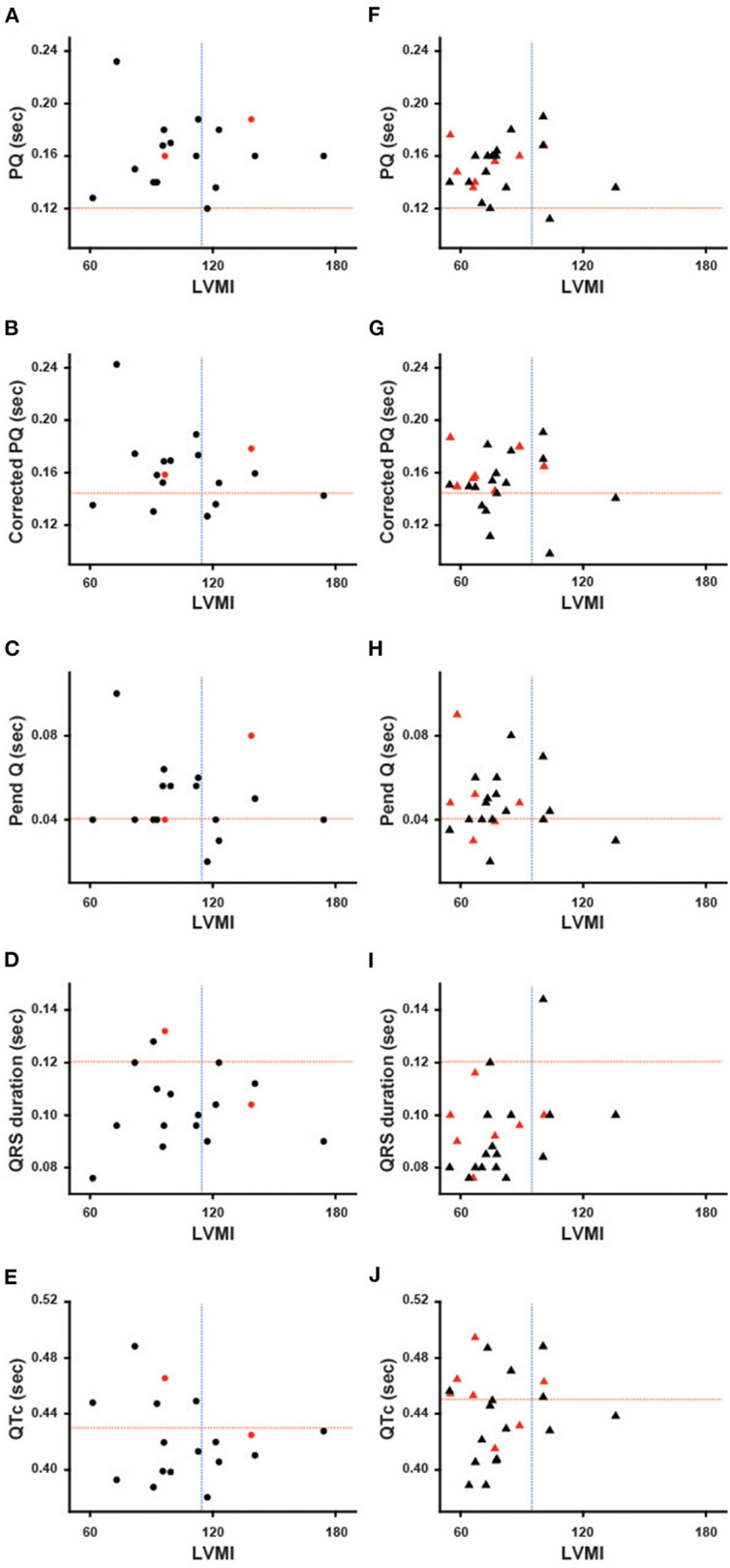
Various ECG parameters plotted against the left ventricular mass index of each patient. The left columns show the parameters of male patients **(A–E)**, and the right columns show the parameters of female patients **(F–J)**. Black symbols show the patients with the classical type, and red symbols show the patients with the late-onset type. The horizontal dotted lines indicate the cut-off value for the abnormalities in each parameter, and the vertical dotted lines indicate the cut-off value for the left ventricular hypertrophy. Corrected PQ, PQ interval divided by the square root of RR interval; Pend-Q, PQ interval minus P-wave duration in lead II; QTc, QT interval divided by the square root of RR interval; LVMI, left ventricular mass index.

Figure 1 shows the ECG parameters in each patient plotted against LVMI. Black symbols indicate the patients with classical type, and red symbols indicate the patients with late-onset type. None of the parameters had any significant correlation with LVMI in male or female patients. Shortening of the PQ interval was found in 3 cases (7.5%) in total (1 of 17 males and 2 of 23 females) (Figures 1A,F). Shortening of the corrected PQ was found in 10 cases (25.0%) in total (5 of 17 males and 5 of 23 females) (Figures 1B,G), and a shortened Pend-Q was found in 19 cases (47.5%) in total (9 of 17 males and 10 of 23 females) (Figures 1C,H). The prolongation of the QRS duration was found in 3 cases (7.5%) in total (2 of 17 males and 1 of 23 females) (Figures 1D,I), and the prolongation of the QTc was found in 17 cases (42.5%) in total (5 of 17 males and 12 of 23 females) (Figures 1E,J). These results indicate that intraventricular conduction was usually unaltered, but the repolarization phase was prolonged in our cohort of Fabry patients.

### The Relevance of ECG Criteria to Evaluate LVH

**Figure 2:**
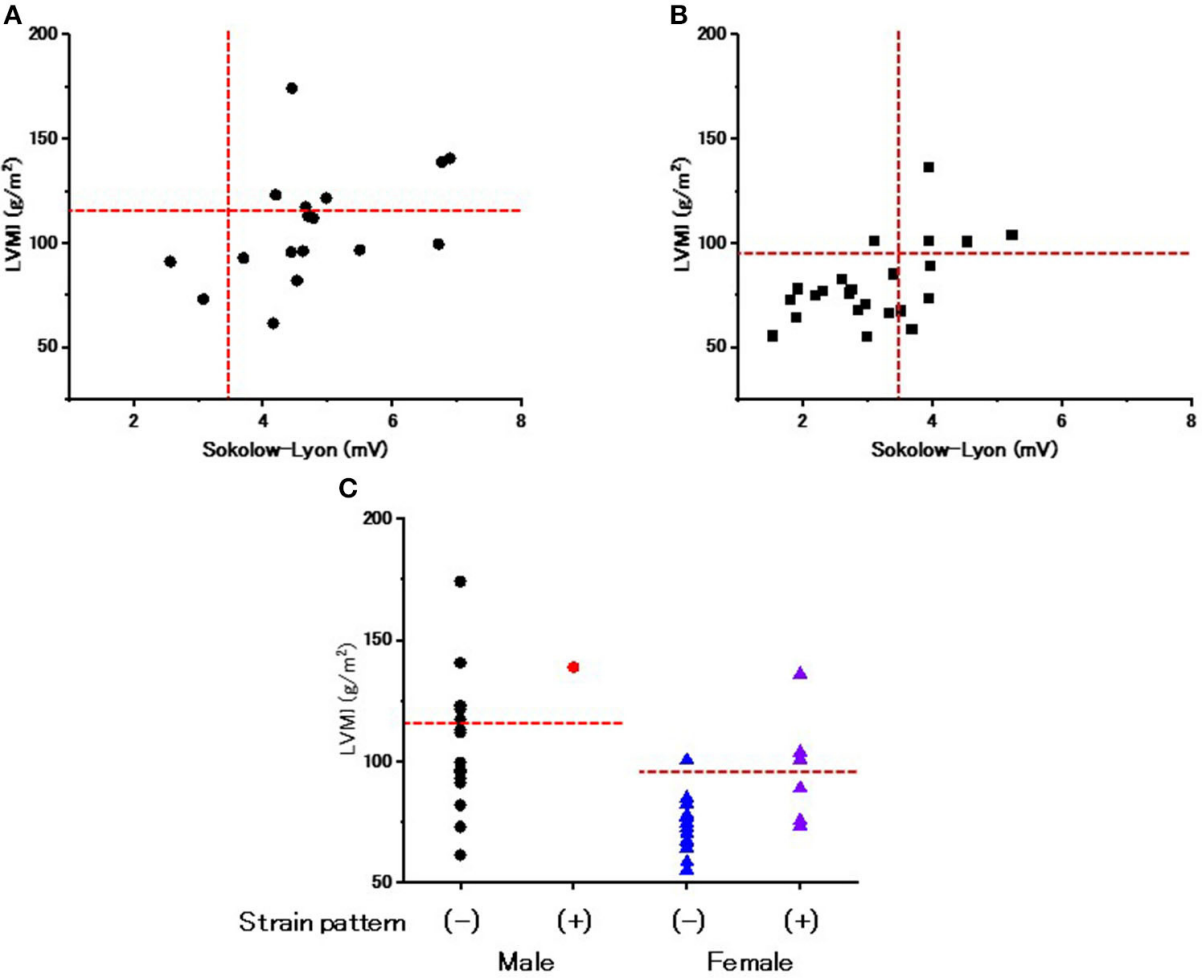
The evaluation of left ventricular hypertrophy and repolarization abnormality on ECGs at the start of enzyme replacement therapy. **(A)** The relationship between the Sokolow-Lyon index and left ventricular mass index (LVMI) evaluated by echocardiography in male patients at the start of enzyme replacement therapy. Horizontal dotted lines indicate the cut-off value of left ventricular hypertrophy by echocardiography. Vertical dotted lines indicate the cut-off value of left ventricular hypertrophy by the Sokolow-Lyon index. **(B)** The relationship between the Sokolow-Lyon index and LVMI evaluated by echocardiography in female patients at the start of enzyme replacement therapy. Horizontal dotted lines indicate the cut-off value of left ventricular hypertrophy by echocardiography. Vertical dotted lines indicate the cut-off value of left ventricular hypertrophy by the Sokolow-Lyon index. **(C)** The LVMI with and without a strain pattern. Dotted lines indicate the cut-off value to determine left ventricular hypertrophy. Closed black circles indicate the male patients without a strain pattern. Closed red circles indicate the male patients with a strain pattern. Blue triangles indicate the female patients without a strain pattern. Purple triangles indicate the female patients with a strain pattern.

Figures 2A,B show the relevance of the use of ECG criteria to evaluate LVH in male and female patients. Using the Sokolow-Lyon index, the sensitivity and specificity for detecting LVH was 100 and 18.2% in male patients and 80 and 77.8% in female patients, respectively. Figure 2C shows the prevalence of the strain pattern at the initiation of ERT. A strain pattern was found 1 (5.9%) male patient but in 7 (30.4%) female patients. Furthermore, 3 of the 7 female patients with a strain pattern did not have LVH on echocardiography (42.9%). These results indicate that a strain pattern is more common in female patients than in male patients, even without overt LVH.

### Changes in Parameters of ECG During Long-Term ERT in Male Patients

**Figure 3:**
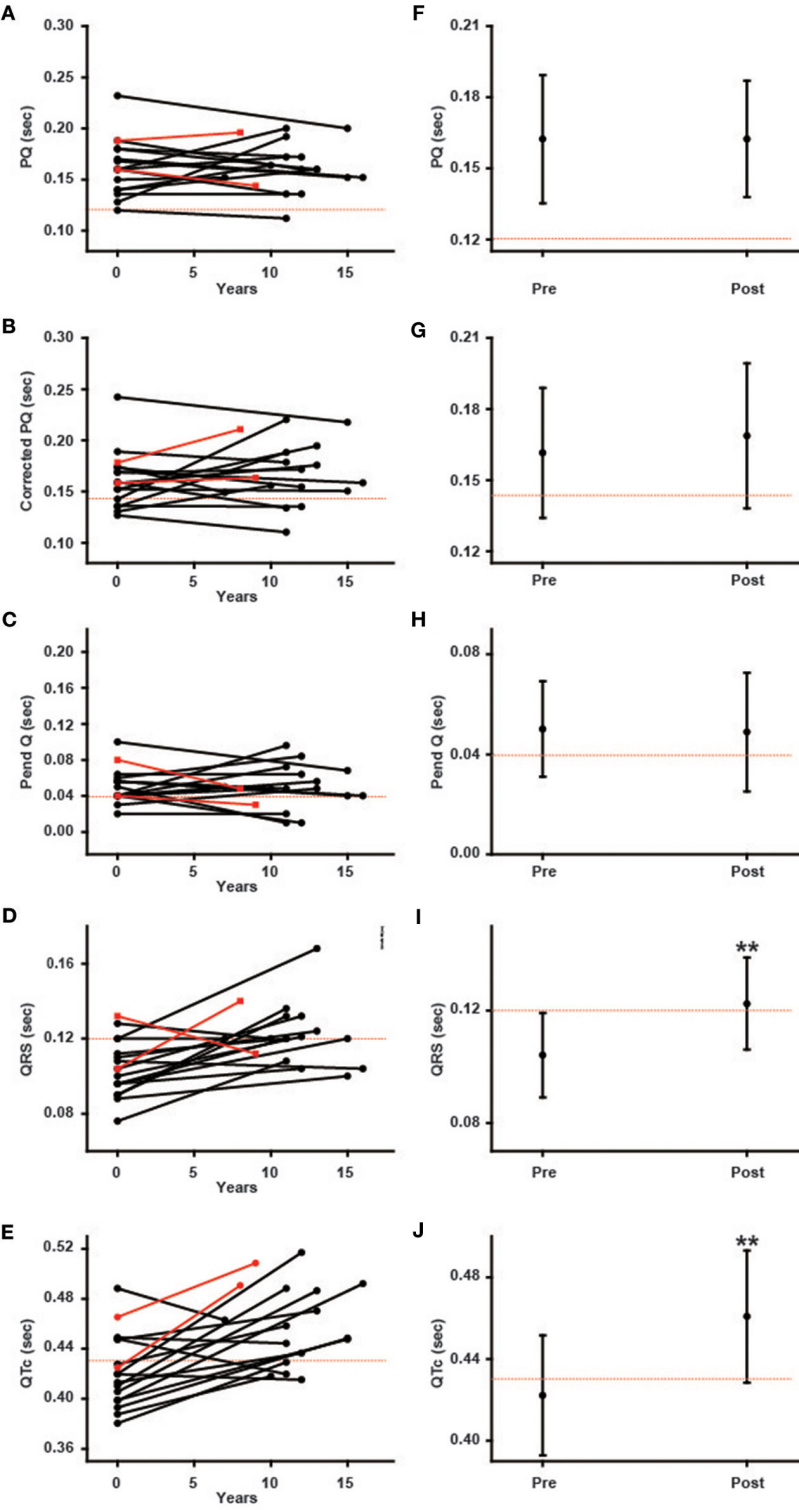
Changes in various ECG parameters during long-term enzyme replacement therapy in male patients. In the left columns, each line indicates the time-dependent changes in ECG parameters in each patient **(A–E)**. The black lines show the patients with the classical type, and the red lines show the patients with the late-onset type. The right columns show the average values of ECG parameters before (Pre) and after (Post) long-term enzyme replacement therapy **(F–J)**. The dotted lines indicate the cut-off value for abnormalities in each parameter. Corrected PQ, PQ interval divided by the square root of RR interval; Pend-Q, PQ interval minus P-wave duration in lead II; QTc, QT interval divided by the square root of RR interval. **p < 0.01 comparison between before (Pre) and after (Post) enzyme replacement therapy.

Figure 3 shows various ECG parameters before and after long-term ERT in male patients. The left columns show the changes in the parameters of each patient, and the right columns show the average values of the ECG parameters. In the left columns, the black lines indicate the patients with the classical type, and the red lines indicate the patients with the late-onset type. The prevalence of the shortening of the PQ interval was unchanged (from 5.9 to 5.9%), and the average PQ interval showed no significant changes (from 162.4 ± 27.1 ms to 162.4 ± 24.5 ms) during long-term ERT (Figures 3A,F). The prevalence of the shortening of the corrected PQ did not change significantly (from 29.4 to 17.6%), and the average corrected PQ showed no significant changes (from 161.6 ± 27.5 ms to 168.8 ± 30.6 ms) during long-term ERT (Figures 3B,G). The prevalence of shortening of the Pend-Q also did not change significantly (from 52.9 to 35.3%), and the average Pend-Q showed no significant changes (from 50.1 ± 19.1 ms to 48.9 ± 23.7 ms) during long-term ERT (Figures 3C,H). The prevalence of the prolongation of the QRS duration did not change significantly (from 11.8 to 41.2%) during long-term ERT, but the average QRS duration was significantly prolonged after ERT (from 104.1 ± 15.1 ms to 122.4 ± 16.4 ms) (Figures 3D,I). There was one male patient who showed right bundle branch block after long-term ERT. The prevalence of the prolongation of the QTc was significantly increased (from 29.4 to 76.5%), and the average QTc was significantly prolonged (from 422.2 ± 29.3 ms to 460.8 ± 32.2 ms) after long-term ERT (Figures 3E,J).

### Changes in Parameters of ECG During Long-Term ERT in Female Patients

**Figure 4:**
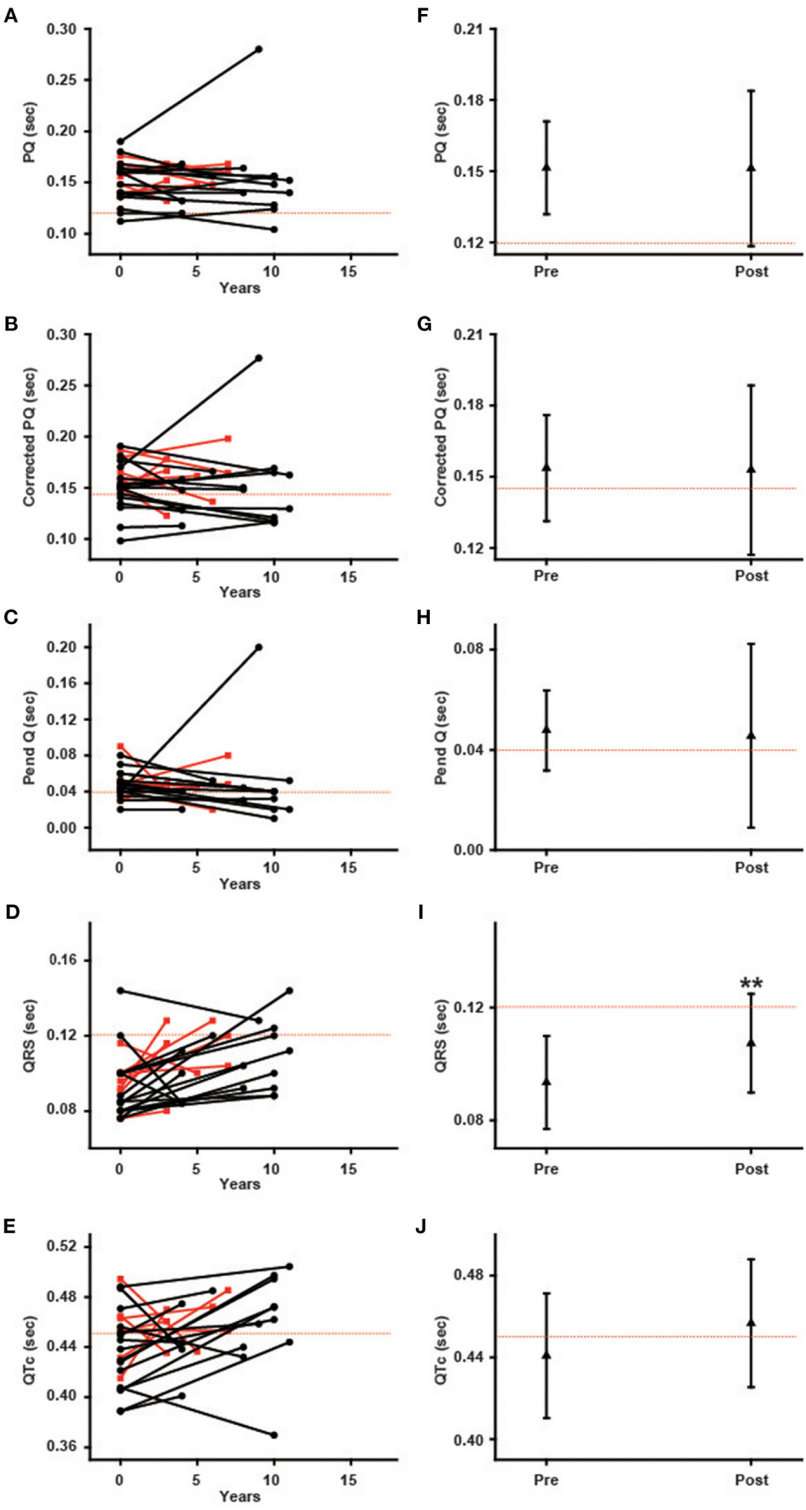
Changes in various ECG parameters during long-term enzyme replacement therapy in female patients. In the left columns, each line indicates the time-dependent changes in ECG parameters in each patient **(A–E)**. The black lines show the patients with the classical type, and the red lines show the patients with the late-onset type. The right columns show the average values of ECG parameters before (Pre) and after (Post) long-term enzyme replacement therapy **(F–J)**. The dotted lines indicate the cut-off value for abnormalities in each parameter. Corrected PQ, PQ interval divided by the square root of RR interval; Pend-Q, PQ interval minus P-wave duration in lead II; QTc, QT interval divided by the square root of RR interval. **p < 0.01 comparison between before (Pre) and after (Post) enzyme replacement therapy.

Figure 4 shows the ECG parameters before and after ERT in female patients. The left columns show the changes in the parameters of each patient, and the right columns show the average values of the parameters. In the left columns, the black lines indicate the patients with the classical type, and the red lines indicate the patients with the late-onset type. The prevalence of shortening of the PQ interval was unchanged (from 8.7 to 8.7%), and the average PQ interval showed no significant changes (from 151.4 ± 19.6 ms to 151.1 ± 32.8 ms) during long-term ERT (Figures 4A,F). The prevalence of shortening of the corrected PQ did not change significantly (from 21.7 to 39.1%), and the average corrected PQ showed no significant changes (from 153.6 ± 22.4 ms to 152.8 ± 35.7 ms) during long-term ERT (Figures 4B,G). The prevalence of shortening of the Pend-Q did not change significantly (from 43.5 to 60.9%), and the average Pend-Q showed no significant changes (from 47.8 ± 16.0 ms to 45.6 ± 36.7 ms) during long-term ERT (Figures 4C,H). The prevalence of the prolongation of the QRS duration did not change significantly (from 4.3 to 21.7%) during long-term ERT, but the average QRS duration was significantly prolonged (from 93.4 ± 16.4 ms to 107.3 ± 17.5 ms) after ERT (Figures 4D,I). There was one female patient with left bundle branch block after long-term ERT. The prevalence of the prolongation of the QTc did not change significantly (from 47.8 to 60.8%), and the average QTc showed no significant changes (from 440.8 ± 30.4 ms to 456.6 ± 31.2 ms) during long-term ERT (Figures 4E,J).

### The Utility of the ECG Criteria for Evaluating LVH After Long-Term ERT

**Figure 5:**
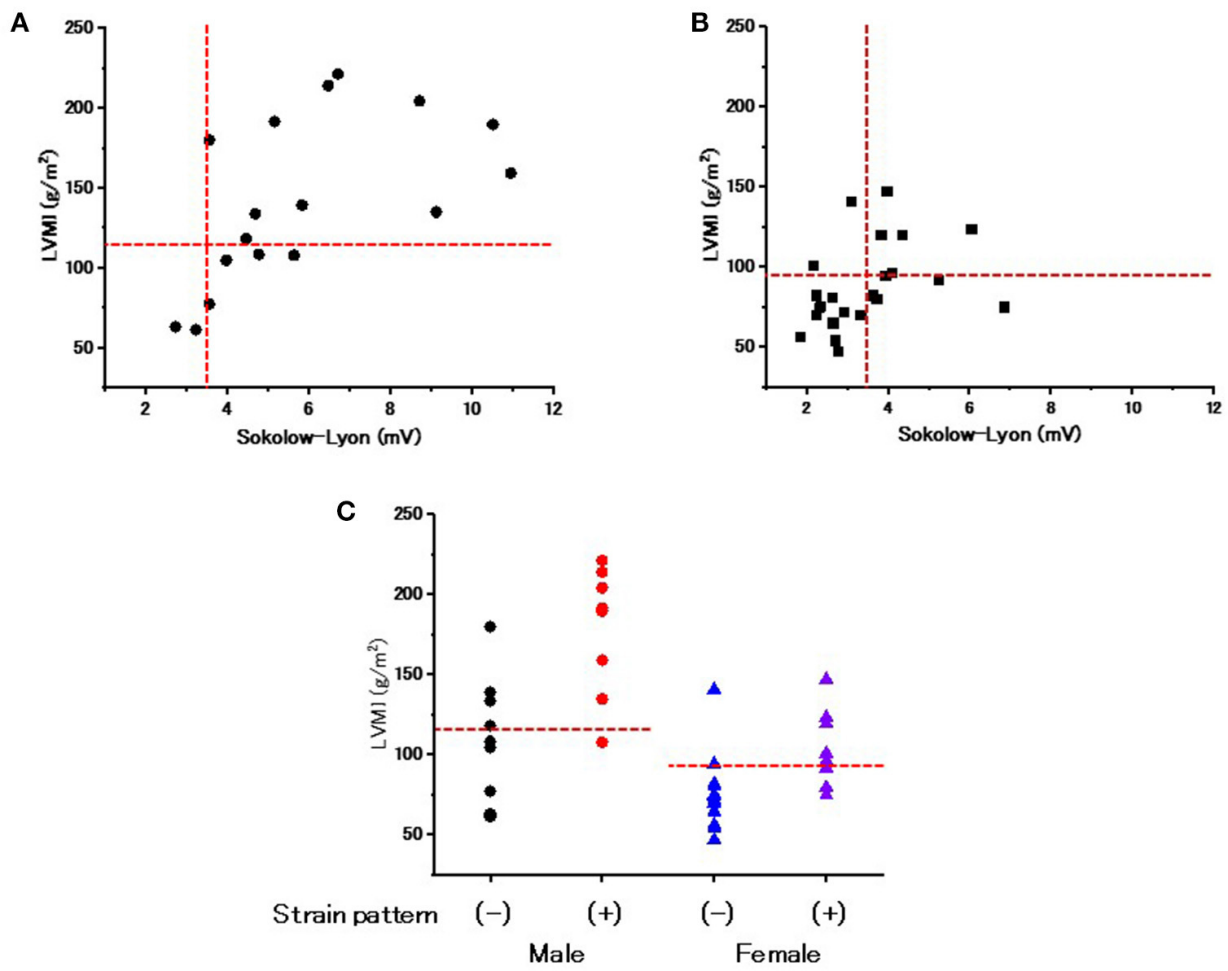
The evaluation of left ventricular hypertrophy and repolarization abnormality on ECGs after long-term enzyme replacement therapy. **(A)** The relationship between the Sokolow-Lyon index and left ventricular mass index (LVMI) evaluated by echocardiography in male patients after long-term enzyme replacement therapy. Horizontal dotted lines indicate the cut-off value of left ventricular hypertrophy by echocardiography. Vertical dotted lines indicate the cut-off value of left ventricular hypertrophy by the Sokolow-Lyon index. **(B)** The relationship between the Sokolow-Lyon index and LVMI evaluated by echocardiography in female patients after long-term enzyme replacement therapy. Horizontal dotted lines indicate the cut-off value of left ventricular hypertrophy by echocardiography. Vertical dotted lines indicate the cut-off value of left ventricular hypertrophy by the Sokolow-Lyon index. **(C)** The LVMI with and without a strain pattern after long-term enzyme replacement therapy. Dotted lines indicate the cut-off value to determine left ventricular hypertrophy. Closed black circles indicate the male patients without a strain pattern. Closed red circles indicate the male patients with a strain pattern. Blue triangles indicate the female patients without a strain pattern. Purple triangles indicate the female patients with a strain pattern.

Figures 5A,B show the use of ECG criteria to evaluate LVH after long-term ERT. Regarding the Sokolow-Lyon index, its sensitivity and specificity for detecting LVH were 100% and 50% in male patients and 71 and 69% in female patients, respectively. Figure 5C shows the prevalence of the strain pattern after long-term ERT, which was significantly increased (from 5.9 to 47.1%) after long-term ERT in male patients. Most male patients with the strain pattern had LVH (87.5%). Regarding female patients, the prevalence of the strain pattern did not change significantly (from 30.4 to 34.8%) during long-term ERT. Among the female patients with a strain pattern, 62.5% had LVH, while 37.5% did not have LVH.

### Subgroup Analyses of the Changes in ECG Parameters During Long-Term ERT

**Figure 6:**
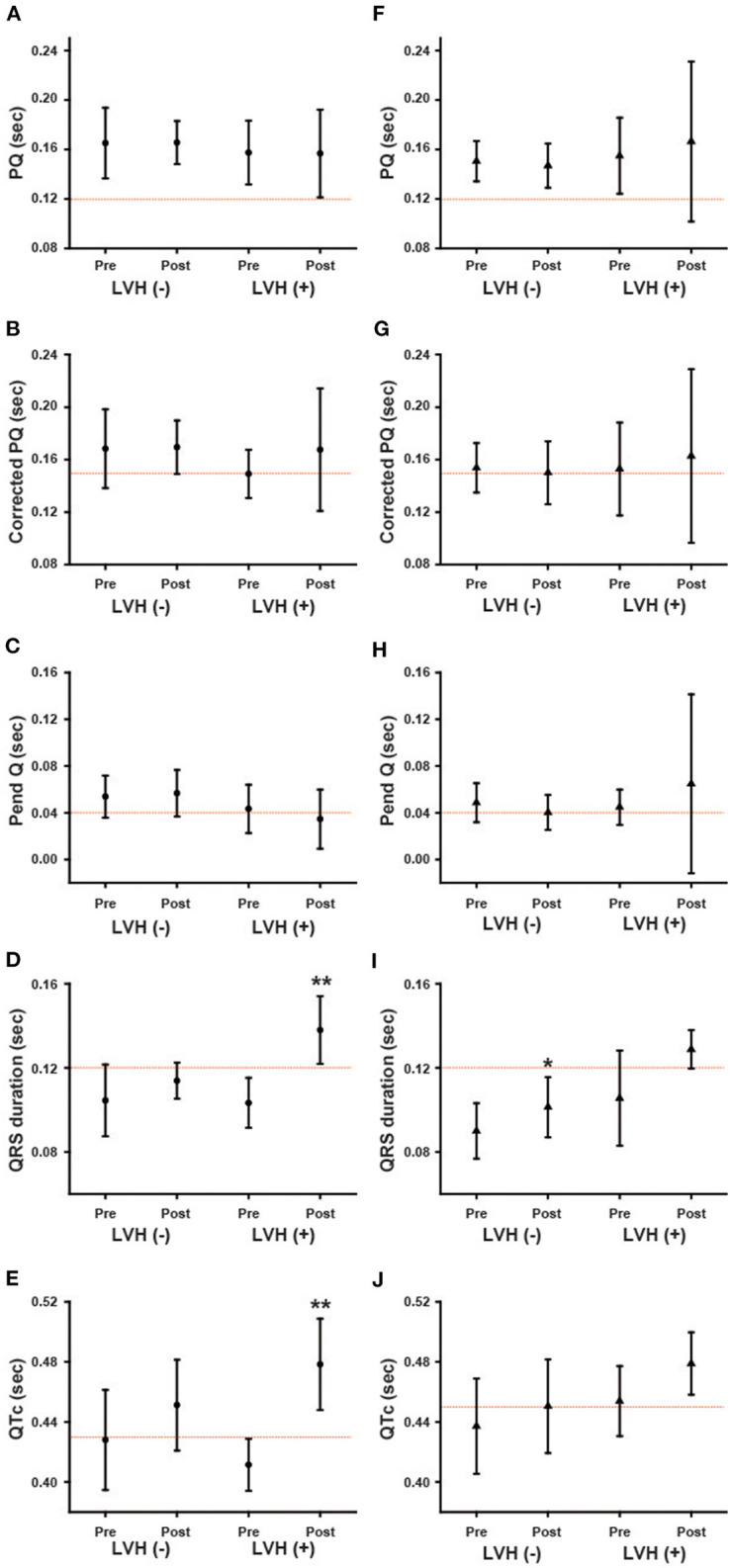
The influence of the left ventricular hypertrophy on the changes in various ECG parameters during long-term enzyme replacement therapy. These figures show the average values of ECG parameters before (Pre) and after (Post) long-term enzyme replacement therapy in the patients without left ventricular hypertrophy [LVH (–)] and in the patients with left ventricular hypertrophy [LVH (+)]. The left columns show the results from male patients **(A–E)** and the right columns show the results from female patients **(F–J)**. The dotted lines indicate the cut-off value for abnormalities in each parameter. Corrected PQ, PQ interval divided by the square root of RR interval; Pend-Q, PQ interval minus P-wave duration in lead II; QTc, QT interval divided by the square root of RR interval. *p < 0.05, **p < 0.01 comparison between before (Pre) and after (Post) enzyme replacement therapy.

We further analyzed the ECG parameters regarding baseline LVMI. Male and female patients were divided into two groups: patients without LVH [LVH (–)] and patients with LVH [LVH (+)]. Figure 6 presents the ECG parameters of the patients in these two different subgroups. The baseline values of each parameter did not differ significantly between the LVH (–) and LVH (+) groups. The left columns show the ECG parameters in male patients, while the right columns show the parameters in female patients. In male patients, the QRS and QTc were significantly prolonged after long-term ERT (Post) compared with the baseline values (Pre) in the LVH (+) group (Figures 6D,E). This result shows that the prolongation of the QRS and QTc in male patients reflects patients with LVH. In contrast, the QRS was significantly prolonged after long-term ERT in the LVH (–) group among female patients (Figure 6I).

**Figure 7:**
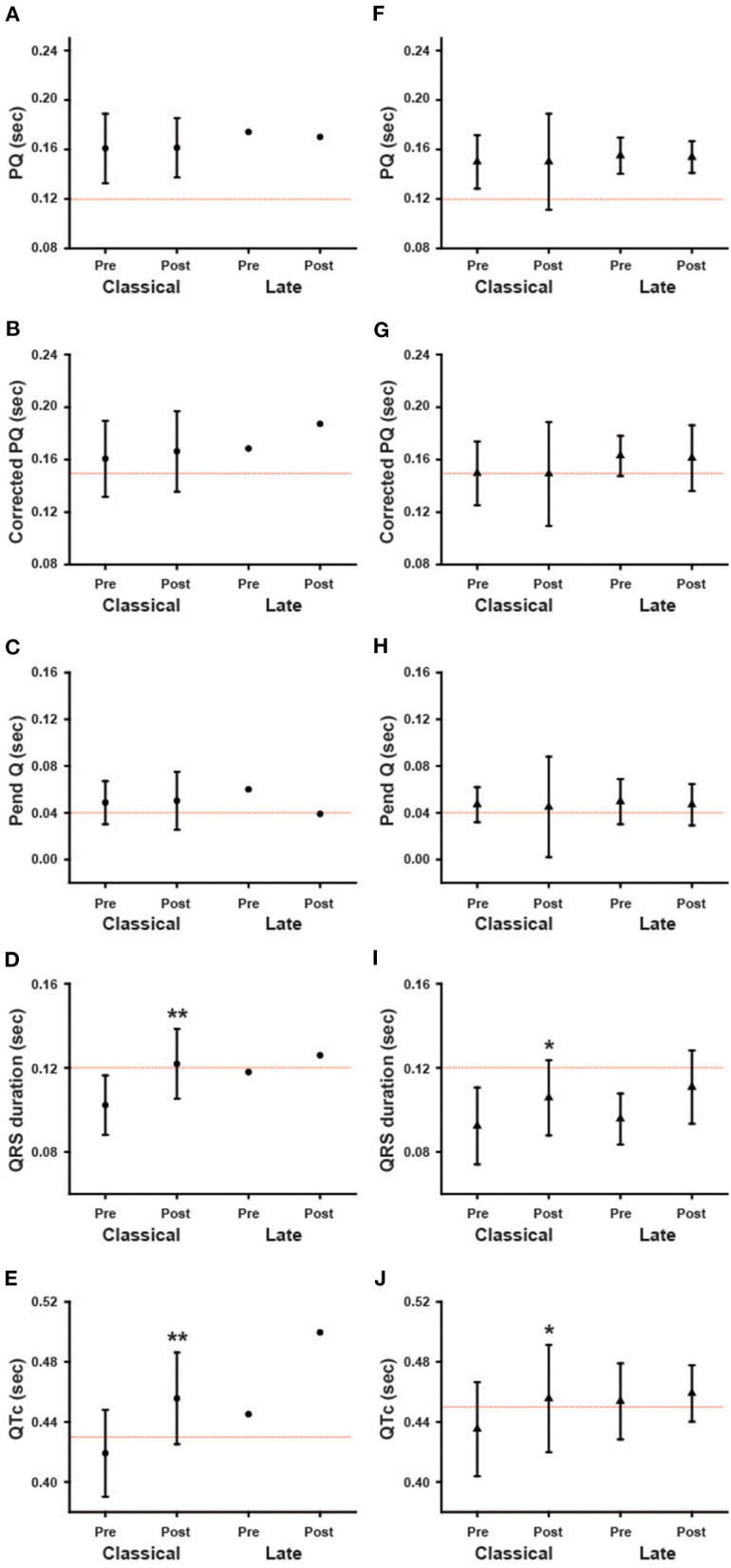
The influence of phenotype/genotype on the changes in various ECG parameters during long-term enzyme replacement therapy. These figures show the average values of ECG parameters before (Pre) and after (Post) long-term enzyme replacement therapy in the patients with classical type (Classical) and late-onset type (Late). The left columns show the results from male patients **(A–E)**, and the right columns show the results from female patients **(F–J)**. *p < 0.05, **p < 0.01 comparison between before (Pre) and after (Post) enzyme replacement therapy.

We also analyzed the ECG parameters regarding phenotype/genotype. Both male and female patients were divided into two groups: patients with the classical type and patients with the late-onset type. Because only two male patients were the late-onset type, we were unable to statistically compare the classical and late-onset types among male patients. Figure 7 shows the ECG parameters for the classical type and late-onset type. None of the parameters at baseline were significantly different between the classical and late-onset types among female patients. In classical type male patients, the QRS and QTc were significantly prolonged following long-term ERT (Post) compared to the baseline values (Pre) (Figures 7D,E). In female patients with the classical type, the QRS and QTc were significantly prolonged after long-term ERT (Post) compared to the baseline values (Pre) (Figures 7I,J). In female patients with the late-onset type, there were no significant differences in the ECG parameters before and after ERT.

## Discussion

### Baseline Analyses of Various Parameters on ECG

Previous reports suggest that the shortening of the PQ interval can be a specific ECG finding in Fabry disease (13). However, a recent report revealed that only 14% of Fabry disease patients showed shortening of the PQ interval (14). Our data showing only a 7.5% prevalence of shortening of the PQ interval was consistent with this finding (Figures 1A,F). We then used the corrected PQ to exclude the influence of bradycardia, which might occur in cases of Fabry disease (17). The prevalence of shortening of the corrected PQ was relatively low (25%) (Figures 1B,G). Therefore, the corrected PQ also cannot be used to detect the early stage of Fabry disease. Another report suggested that the shortening of the P wave duration was specific for Fabry disease (20), however, we found only two female patients who showed shortening of the P wave duration (data not shown). Nearly half of the patients (47.5%) showed shortening of the Pend-Q (Figures 1C,H), which is an index of the PQ interval excluding the P wave duration (17). Therefore, the Pend-Q is an ideal parameter of atrio-ventricular conduction and can reflect the acceleration of atrio-ventricular conduction in the early phase of Fabry disease.

The QRS duration is an index of intraventricular conduction and has been reported to be correlated with the LVMI (21). The average value of the QRS duration was significantly longer in male patients than in female patients, despite their younger average age (Table 2), which might reflect a higher LVMI in male patients than in female patients. Abnormalities of the repolarization phase on ECGs have also been reported (20) and may be related to myocardial fibrosis in Fabry disease (21). In addition, the reduced probability of the prolongation of the QTc may be used to differentiate Fabry disease from cardiac amyloidosis (17). However, the prolongation of the QTc was not rare, being detected in 42.5% of the patients (especially, more than half in female patients) in our cohort of Fabry disease (Figures 1E,J). The strain pattern, another candidate of repolarization abnormality, was detected in only 5.9% of male patients, but in 30.4% of female patients (Table 2). Furthermore, nearly half of the female patients with a strain pattern did not show LVH (Figure 2C). In addition, the average QTc value tended to be longer in female patients than in male patients (Table 1). The more frequent appearance of the strain pattern and longer QTc might suggest that repolarization abnormalities appear early in female patients, even among those without overt LVH. The strain pattern and the prolongation of the QTc can reflect the early appearance of myocardial fibrosis (which can trigger various arrhythmias) detected by the late gadolinium enhancement on cardiac magnetic resonance imaging in female patients, even those with mild LVH (8) or no LVH (22, 23).

An early diagnosis is important for introducing ERT in Fabry patients, and an ECG is effective as a modality in terms of its simplicity. At the baseline analysis of the ECG parameters, shortening of the Pend-Q is a specific finding in Japanese Fabry patients, and a strain pattern without LVH in female patients can be considered indicative of Fabry disease.

### Time-Dependent Changes in the ECG Parameters During ERT

In both male and female patients, the prevalence and average value of the PQ interval, corrected PQ and Pend-Q did not change significantly during long-term ERT (Figures 3, 4). Some reports have shown that the shortened PQ interval improved during ERT (24, 25), however, others have shown that the PQ interval was not altered during ERT (26). Since the PQ interval is considered to be prolonged with age-dependent progression in Fabry disease (27), a lack of the changes in the average values of the parameters of atrio-ventricular conduction (PQ interval, corrected PQ, and Pend-Q) might suggest the slowing of atrio-ventricular conduction disturbance during ERT in Japanese Fabry patients.

The average QRS duration and QTc were significantly prolonged in male patients during long-term ERT (Figures 3I,J), while only the average QRS duration was significantly prolonged in female patients during long-term ERT (Figure 4I). A subgroup analysis revealed that the prolongation of the QRS duration and QTc in male patients reflected the results obtained from male patients with LVH (Figures 6D,E). Another subgroup analysis revealed the significant prolongation of the QRS duration and QTc only in those with the classical type mutation among female patients (Figures 7I,J). Because the average QTc in female patients tended to be longer than that in male patients before ERT, the average QTc after ERT was almost the same between male and female patients (458.9 ± 29.6 ms vs. 456.6 ± 31.2 ms). After long-term ERT, the prevalence of a strain pattern was significantly increased in male patients (Figure 5C). Again, because the prevalence of a strain pattern in female patients showed a higher tendency than that in male patients before ERT, the prevalence of a strain pattern after ERT was similar between male and female patients (47.1 vs. 34.8%). The prolongation of the QRS duration and QTc are not specific findings of Fabry disease and are considered to be related to LVH and myocardial fibrosis (21). Therefore, the time-dependent changes in QRS duration and QTc might indicate the progression of myocardial damage in male patients with LVH and in female patients with a classical type mutation during long-term ERT.

In conclusion, the present results show that the shortening of the Pend-Q in both genders and a strain pattern without LVH in female patients can be considered indicative of Fabry disease. The prolongation of the QRS duration and QTc indicates the gradual progression of myocardial damage, even during long-term ERT, especially in male patients with LVH and in female patients with the classical type mutation.

### Limitations

Several limitations associated with the present study warrant mention. This study was a retrospective single-center observational study. Because of the rare disease nature of Fabry disease, the sample size is small. In our cohort, the type of ERT and the type of phenotype/genotype were heterogeneous. Our indication of ERT for female patients differs from that described in the national guideline of Fabry disease. Because we had previously determined there to be some merit in starting ERT early, even in female patients, though our own studies, we started ERT at a relatively early point for female patients. Many patients used both types of ERT during long-term follow up. Therefore, we were unable to compare the ECG parameters among patients receiving different types of ERT. We analyzed the ECG data of Fabry patients who were followed at Jikei University Hospital at cross-sectional time points. The duration of ERT treatment was significantly longer in male patients than that in female patients, which might have affected the changes in the ECG parameters. Because of the retrospective nature of the study, we did not perform magnetic resonance imaging or strain imaging echocardiography to evaluate myocardial damage in all patients.

## Data Availability Statement

The original contributions presented in the study are included in the article/supplementary materials, further inquiries can be directed to the corresponding author/s.

## Ethics Statement

The studies involving human participants were reviewed and approved by the Ethics Committee of the Jikei University School of Medicine. The patients/participants provided their written informed consent to participate in this study.

## Author Contributions

SM, TS, MY, and KH conceived of the presented idea and designed the study. SM performed the analysis and took the lead in the writing the manuscript. SM, AN, EF, IA, and MKa performed data collection. KS, MKo, HK, HI, TO, and YE supervised the study. KH and TO edited and revised the manuscript. All authors discussed the results, contributed to the final manuscript, and approved the final version of the manuscript.

## Conflict of Interest

KH, HI, and YE received research funding from Sanofi Genzyme Corporations and Sumitomo Dainippon Pharma. MY received research funding from Dainippon Sumitomo Pharma. TO received research funding from Sanofi Genzyme Corporations, Sumitomo Dainippon Pharma and Amicus Therapeutics, and conducted joint research with JCR Pharmaceuticals. The remaining authors declare that the research was conducted in the absence of any commercial or financial relationships that could be construed as a potential conflict of interest.
